# Perioperative neurocognitive disorder in colorectal cancer surgery: a systematic review of incidence, mechanisms, and interventions

**DOI:** 10.3389/fsurg.2025.1698597

**Published:** 2025-11-18

**Authors:** Xiujin Huang, Zongjie Quan, Chenyang Zhan, Bhushan Sandeep, Jun Bu

**Affiliations:** 1 Department of General Surgery, School of Clinical Medicine, North Sichuan Medical College, Nanchong, Sichuan, China; 2 Department of General Surgery, Chengdu Second People’s Hospital, Chengdu, Sichuan, China; 3 Department of Cardio-Thoracic Surgery, Chengdu Second People’s Hospital, Chengdu, Sichuan, China

**Keywords:** perioperative neurocognitive disorder, postoperative delirium, postoperative cognitive dysfunction, colorectal cancer surgery, systematic review

## Abstract

**Background:**

Perioperative neurocognitive disorder (PND) represents a significant impediment to postoperative recovery in patients undergoing colorectal cancer surgery, particularly among the elderly. This systematic review synthesizes contemporary evidence on PND incidence, risk stratification, and prophylactic strategies.

**Methods:**

Adhering to PRISMA guidelines, 343 records were identified from PubMed, Embase, and Web of Science. Bibliometric profiling delineated influential journals, institutions, and seminal references. Following deduplication and screening, 11 randomized controlled trials (RCTs) met inclusion criteria for systematic analysis.

**Results:**

Bibliometric analysis revealed Journal of Geriatric Oncology (impact factor 2.7) and Amphia Hospital (Netherlands) as top contributors. PND incidence ranged from 3.4% to 56% across studies. Key risk factors included advanced age (mean 63–76 years), ASA status II–III, and open surgery. Prophylactic interventions reduced PND: melatonin decreased POD by 20%; dexmedetomidine reduced PND by 10.8%–25%. Fast-track surgery lowered POD by 9.5%. Mechanistically, effective strategies attenuated neuroinflammation (IL-6/TNF-α reduction) and optimized analgesia.

**Conclusions:**

Multimodal interventions—especially dexmedetomidine-enhanced analgesia and fast-track protocols—consistently mitigate PND. Standardization of PND assessment tools and diversification of study populations are urgently needed.

## 1 Introduction

Colorectal cancer remains a globally prevalent malignancy, with surgical resection as its foundational treatment (1, 2). Despite advances in surgical techniques and perioperative care, colorectal cancer surgery poses unique challenges, especially in elderly patients. Characterized by distinct physiological and psychological profiles, this population faces greater complexity in surgical adaptability and postoperative recovery. Major procedures like colorectal resection carry inherent risks and long-term impacts on elderly patients’ recovery and quality of life, with perioperative neurocognitive disorder (PND) being a critical complication (3, 4).

PND is a clinically significant central nervous system complication predominantly affecting patients aged ≥60 years undergoing major surgery (5). This umbrella term encompasses acute-to-chronic neurocognitive impairments (within 12 months postoperatively), including postoperative delirium (POD), postoperative cognitive dysfunction (POCD), and delayed neurocognitive recovery (DNR) (5). Its core pathophysiology involves disturbed consciousness, circadian rhythm disruption, and psychomotor dysfunction (5). Beyond hindering Enhanced Recovery After Surgery (ERAS) protocols, PND independently increases all-cause mortality and imposes substantial socioeconomic burdens via prolonged hospitalization and rehabilitation costs (6, 7).

Elderly colorectal cancer surgery patients are particularly susceptible to PND due to prevalent malnutrition, sarcopenia, and frailty (8–12). A recent meta-analysis of 17 studies (4,472 patients) identified psychiatric history, transfusion, comorbidities, male gender, and advanced age as significant POD predictors (13). This aligns with Lee et al., who demonstrated hypoalbuminemia and prior delirium episodes as independent POD predictors (14). Additionally, a prospective nested case-control study (443 patients) by Cui et al. linked elevated postoperative pain, limited education, and sleep disruption to POD pathogenesis (15). Emerging neuroprotective strategies, such as dexmedetomidine-augmented transvs. abdominis plane blocks, reduce POCD incidence (7). However, comprehensive mapping of mechanistic evolution and research trends remains lacking.

To address this gap, we conducted a bibliometric analysis spanning 2006–2025 to explore research trends and knowledge hotspots in PND among colorectal cancer surgery patients. This analysis maps global research productivity and collaboration networks, identifies key themes and emerging topics, traces knowledge evolution, and provides clinical implications and future directions.

## 2 Methods

### 2.1 Search strategy

This study was performed according to the criteria of the Preferred Reporting Items for Reviews articles and Meta-Analyses (PRISMA) statement (16). We searched electronic databases including Pubmed, Embase and the Web of Science citation index from inception to July 2025 for articles meeting the listed inclusion criteria (in investigating the PND in colorectal cancer surgery). The following terms were selected according to the patient, intervention, outcomes and surgery (PICOS) framework, the following terms were selected: “perioperative neurocognitive disorders,” OR “postoperative delirium,” OR “postoperative cognitive dysfunction”,

AND “colorectal cancer surgery,” with Boole logic. We also screened references for the identified articles. There were no language restrictions on searching for articles (search strategy was combined with the terms detailed in the Supplementary Appendix).

### 2.2 Study selection

#### 2.2.1 Bibliometric analysis

Publications were systematically retrieved from the Web of Science Core Collection (WoSCC), incorporating both the Science Citation Index Expanded (SCI-EXPANDED; 1900-present) and Social Sciences Citation Index (SSCI; 1998-present). This database was selected for its comprehensive coverage of high-impact evidence in biomedical research. The plain text format derived from WoSCC can be directly used for visualization with Platform https://smartdata.las.ac.cn/SciExplorer/ and R software. To minimize bias during literature retrieval, data inclusion, and deduplication, at least two researchers independently conducted the literature search and inclusion process. In cases of disagreement, a third researcher was consulted for judgment. Additionally, all included publications were retrieved from the database on the same day. A total of 147 studies from the Web of Science database were identified for bibliometric analysis. First, publication statistics and research distribution were generated using literature data governance and analysis system (LDGAS) from SciExplorer; second, knowledge trends, collaboration authors, regions, organizations, and keywords was generated using knowledge matrix visualization system (KMVS) from SciExplorer. Third, R software was used to illustrate the top ten most influential journals, institutions, and the global citation of reference. That analysis covered different types of studies such as basic research, clinical randomized controlled trials, clinical cohort studies, clinical case-control studies, reviews (both narrative and systematic), and meta-analyses.

#### 2.2.2 Systematic review

Following retrieval from three databases, all records underwent deduplication using EndNote reference management software. For the systematic review, inclusion criteria were defined as follows: (1) the articles had to be published in English and be full-length articles; (2) case reports, protocols, letters, reviews and meta-analyses, conference abstracts, ongoing study and observational studies were excluded; (3) only RCTs with complete data were included. (4) The intervention arm had to investigate the correlation of PND in patients undergoing colorectal cancer surgery; and (5) the outcomes had to include the incidence of PND.

### 2.3 Data extraction

Two investigators independently performed data extraction using a standardized protocol. All extracted variables were recorded in a structured Microsoft Excel spreadsheet (Office 2021). The following characteristics of all included trials were collected: publication year, name of the first author, country, age of participants, type of surgery, PND incidence, and conclusions.

### 2.4 Risk of bias assessment

For the RCTs included in the systematic review, we applied the revised JBI Critical Appraisal Tool for Randomized Controlled Trials to evaluate the risk of bias (17). This tool assesses risk of bias across seven core dimensions, included random sequence generation; allocation concealment; blinding of participants, researchers, and outcome assessors; management of incomplete outcome data; selective outcome reporting; baseline equivalence between the intervention and control groups; and other potential sources of bias. Each included RCT was assigned an overall risk of bias rating (low, unclear, or high) based on the integrated assessment of these seven dimensions.

## 3 Results

**Figure 1:**
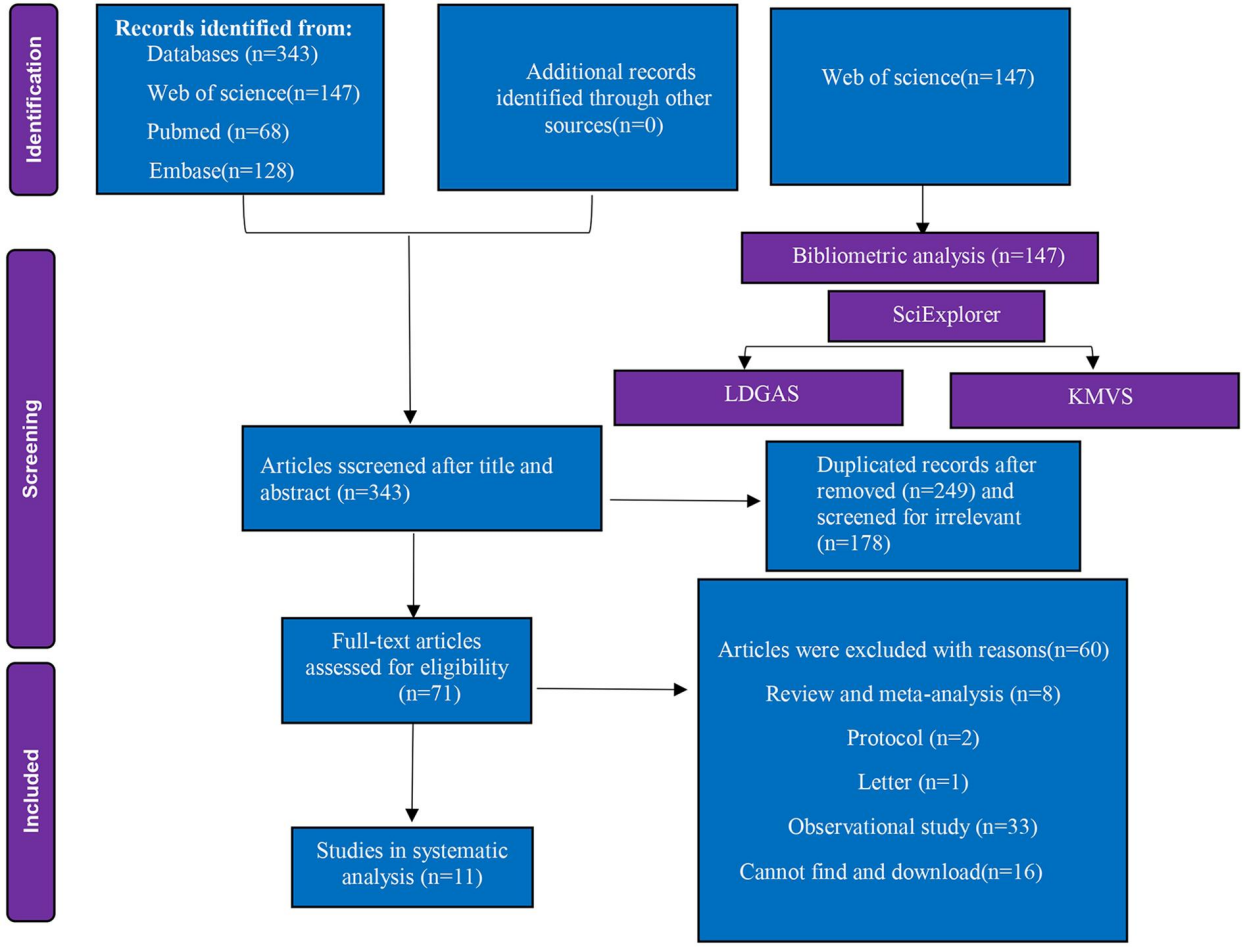
Flow diagram of included and excluded studies.

The study selection process and flow chart, as delineated in Figure 1. Initial database interrogation yielded 343 unique citations. Of these, 147 publications derived from Web of Science underwent bibliometric profiling. Following full-text screening and application of inclusion criteria, 11 RCTs encompassing 1,308 patients (recruited between 2014 and 2025) were retained for systematic analysis.

### 3.1 Bibliometric results

#### 3.1.1 Global publication distribution

**Figure 2:**
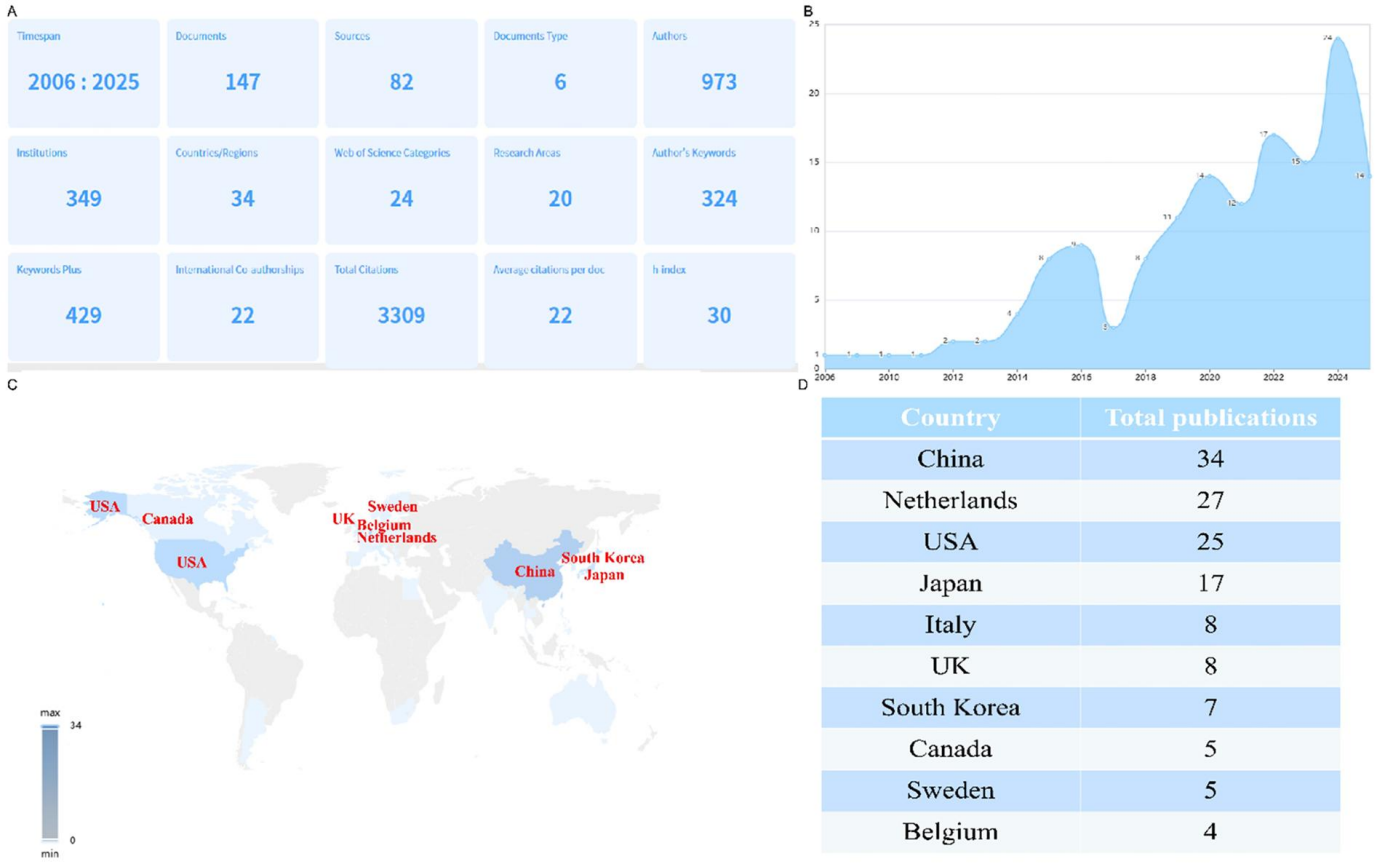
Global publication distribution on perioperative neurocognitive disorders in patients undergoing colorectal cancer surgery. **(A)** Overview of publication metrics; **(B)** annual publication trends; **(C)** country-level contributions; **(D)** rank countries by publication volume.

Figure 2A provides a comprehensive summary of research output related to PND in colorectal cancer surgery from 2006 to 2025. Key insights include: 147 documents were published across 82 sources (journals), involving 973 authors from 349 institutions. This indicates a moderately active but growing field with broad collaboration. Research spans 34 countries/regions, with 22 international co-authorships, suggesting global interest but room for more cross-border collaboration. The 3,309 total citations and h-index of 30 reflect significant scholarly influence. The average of 22 citations per document highlights consistent relevance. 324 author keywords and 429 keywords plus reveal diverse terminology, possibly reflecting evolving definitions of PND (e.g., POD/POCD). Furthermore, annul publication trends demonstrated that minimal activity in 2006–2012 (0–5 publications/year), suggesting PND in colorectal surgery was not a focal area; steady increase in 2014–2020 (12–17 publications/year), likely driven by heightened awareness of PND and aging surgical populations; a surge to 24 publications in 2022–2024, possibly linked to updated diagnostic criteria (e.g., DSM-5's “neurocognitive disorders”) or studies on anesthesia techniques (e.g., propofol vs*.* volatile agents) as shown in Figure 2B. In addition, the Figures 2C,D revealed the countries contribution to this area: China leads (*n* = 34), reflecting its robust research investment and large patient populations; Netherlands (*n* = 27) and USA (*n* = 25) follow, indicating strong Western engagement, possibly due to geriatric surgery initiatives; Japan (*n* = 17), Italy/UK (*n* = 8 each), and South Korea (*n* = 7) show regional focus but fewer collaborations.

#### 3.1.2 Research distribution and influential journals/institutions

**Figure 3:**
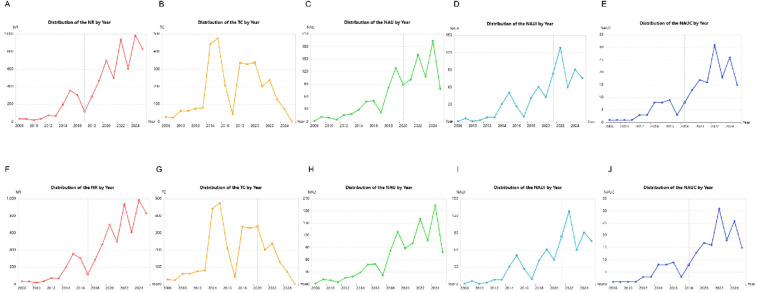
Research distribution on the perioperative neurocognitive disorders in patients undergoing colorectal cancer surgery. **(A)** Distribution of the number of reference (NR) by year; **(B)** distribution of the total citations (TC) by year; **(C)** distribution of the number of authors (NAU) by year; **(D)** distribution of the number of authors’ institutions (NAUI) by year; **(E)** distribution of the number of authors’ regions/countries (NAUC) by year; **(F)** distribution of the number of reference (NR) by frequency; **(G)** distribution of the total citations (TC) by frequency; **(H)** distribution of the number of authors (NAU) by frequency; **(I)** distribution of the number of authors’ institutions (NAUI) by frequency; **(J)** distribution of the number of authors’ regions/countries (NAUC) by frequency.

We conducted research distribution on the PND in colorectal cancer surgery, revealing that from 2006 to 2024 the number of references, total citations, number of authors, number of authors' institutions, and number of authors' regions/countries by year (Figures 3A–E) has dramatically increased, which may reflect the increasing research enthusiasm in this field, the increase in new discoveries. However, the core trend is the turning point of 2014–2016, which needs to be explained in the context of clinical context: this is the critical period when the DSM-5 redefines “POCD” as “PNDs”, and the concept of ERAS is popularized in colorectal cancer surgery. The peak in 2022 may reflect three factors: post-COVID-19 research recovery, increased surgeries for elderly colorectal cancer, and anesthesia neurotoxicity controversies (such as the explosion of research on the effects of sevoflurane on tau protein). Figures 3F–J revealed a typical long-tail distribution of academic citations, which confirms the law of “a small number of literatures dominates the number of citations”. In this filed, it is necessary to focus on the research direction of high-citation literature and encourage the exploration of potential innovations in low-citation literature (such as emerging technologies or interdisciplinary applications).

Subsequently, Table 1 lists the 10 most globally influential journals in the field of PND in the colorectal cancer surgery research and shows the academic performance of each journal through multi-dimensional indicators. 40% (*n* = 4) of United States journals, 30% (*n* = 3) of England journals, and left 3 journals from Netherland (*n* = 1), New Zealand (*n* = 1), and Germany (*n* = 1) respectively. Furthermore, the top journals (*Journal of Geriatric Oncology*, *Annals of Surgical Oncology*) highlight the intersection of aging, cancer, and surgery; 7/10 journals specialize in surgery/oncology (e.g., *Colorectal Disease*, *Surgical Endoscopy*), reflecting the clinical context of PND; only one anesthesiology journal, suggesting limited engagement from anesthesia-focused journals. In addition, *PLoS One* (Rank 4) has the highest average citations (5.88) despite moderate output (*n* = 5), indicating influential interdisciplinary studies. However, *Journal of Geriatric Oncology* (Rank 1) leads in publications (*n* = 11) but has lower average citations (2.73). The dominance of surgical oncology reinforces PND as a postoperative complication rather than a neurobiological disorder, potentially limiting funding for mechanistic work. Table 2 revealed the impact of PND on the academic output of the world's top 10 research institutions in the field of colorectal cancer surgery. Seven of ten institutions are from the Netherlands (led by Amphia Hospital with 22 publications and 98 citations), University of Osaka (Rank 2, 14 publications), Seoul National University (Rank 5, 9 publications), and Southwest Medical University (Rank 9, 7 publications). Furthermore, Netherlands' hospital-university nexus (Amphia, Leiden, Haga) sets the gold standard for clinically embedded research. Asian institutions must convert output into impact via international collaboration and methodological rigor. Include North American institutions (e.g., Mayo Clinic, Johns Hopkins) to leverage their neuroanesthesia expertise.

**Table 1:** The top 10 influential journals on the perioperative neurocognitive disorder in patients undergoing colorectal cancer surgery.

Rank	Journal	Total publications	Total citations	Average citations	Country	Impact factors (2025)
1	Journal of Geriatric Oncology	11	30	2.73	Netherland	2.7
2	Annals of Surgical Oncology	7	11	1.57	United states	3.5
3	Colorectal Disease	6	5	0.83	England	2.7
4	PLoS One	5	29	5.880	United states	2.6
5	World Journal of Gastrointestinal Surgery	5	1	0.20	United states	1.7
6	Surgical Endoscopy and Other Interventional Techniques	4	16	4.00	United states	2.7
7	Ejso	4	9	2.25	England	2.9
8	Clinical Interventions in Aging	4	5	1.25	New Zealand	3.7
9	BMC Anesthesiology	4	3	0.75	England	2.6
10	International Journal of Colorectal Disease	3	17	5.67	Germany	2.3

**Table 2:** The top 10 influential institutions on perioperative neurocognitive disorder in patients undergoing colorectal cancer surgery.

Rank	Institution	Total publications	Total citations	Average citations	Country
1	Amphia hospital	22	98	4.45	Netherlands
2	University of Osaka	14	40	2.86	Japan
3	Leiden University	11	28	2.55	Netherlands
4	University of Groningen	10	21	2.10	Netherlands
5	Seoul National University	9	0	0.00	South Korea
6	Haga hospital	8	34	4.25	Netherlands
7	Diakonessen Hospital	8	25	3.13	Netherlands
8	Utrecht University	7	6	0.86	Netherlands
9	Southwest Medical University	7	4	0.57	China
10	University Medical Center Utrecht	6	12	2.00	Netherlands

#### 3.1.3 Most cited reference and collaboration network

Table 3 demonstrated the top 10 most-cited references in PND research for colorectal cancer surgery. These studies have several obvious characteristics (10, 20–25): first, the focus is highly focused on POD, and 7 out of 10 studies directly study POD (10, 20–23); second, clinical observational research designs (*n* = 10) are generally adopted, and interventional studies are lacking; Third, the main conclusions point to the importance of preoperative assessment, especially the comprehensive assessment of the elderly (CGA) and nutritional status (24, 26). It is worth noting that two studies by the Netherlands team Janssen (60 and 43 citations, respectively) (21, 23), proposed a multimodal prehabilitation regimen is a rare intervention study, showing that prehabilitation can reduce the incidence of delirium (23). Two studies (60 and 49) by the Japanese team Tei emphasized the role of nutritional assessment and minimally invasive surgery in preventing POD (10, 20). The citation distribution reflects three research hotspots: preoperative risk assessment (CGA application) (24, 26), biomarkers such as sarcopenia (Mosk study) (22), and the impact of complications on survival (Breugom study) (25).

**Table 3:** The top 10 most global cited reference on the perioperative neurocognitive disorder in patients undergoing colorectal cancer surgery.

Study	Country	Reference	DOI	Total citation	Types of articles	Conclusions
Raats et al. (2015) (18)	Italy	Risk Factors and Outcomes for Postoperative Delirium after Major Surgery in Elderly Patients	10.1371/journal.pone202.0136071	202	Clinical study	POD is a frequent complication after major surgery in elderly patients and is related to an increase in adverse events, length of hospital stays, and mortality
Patti et al. (2011) (19)	Italy	Risk factors for postoperative delirium after colorectal surgery for carcinoma	10.1016/j.ejon.2011.01.004	62	Clinical study	These findings suggest that POD is a frequent complication after colorectal surgery for carcinoma.
Tei et al. (2010) (20)	Japan	Risk factors for postoperative delirium in elderly patients with colorectal cancer	10.1007/s00464-010-0911-7	60	Clinical study	Preoperative evaluation of nutritional status is important in elderly patients with colorectal cancer in order to prevent POD
Janssen, Steyerberg, Faes (2019) (21)	Netherlands	Multimodal prehabilitation to reduce the incidence of delirium and other adverse events in elderly patients undergoing elective major abdominal surgery: An uncontrolled before-and-after study	10.1371/journal.pone.0218152	60	Clinical study	The current prehabilitation program is feasible and safe, and can reduce delirium incidence in elderly patients undergoing elective major abdominal surgery
Mosk et al. (2018) (22)	Netherlands	Low skeletal muscle mass as a risk factor for postoperative delirium in elderly patients undergoing colorectal cancer surgery	10.2147/CIA.S175945	50	Retrospective observational cohort study	Age, history of delirium, LSMM, and malnourishment or physical dependency were independently associated with POD
Tei et al. (2016) (10)	Japan	Incidence and risk factors of postoperative delirium in elderly patients who underwent laparoscopic surgery for colorectal cancer	10.1007/s00384-015-2335-2	49	Clinical study	The results suggest that the risk of POD is associated with older age, past history of delirium or dementia, operative approach, Organ/Space SSI
Janssen, Steyerberg, Langenberg (2019) (23)	Netherlands	Risk factors for postoperative delirium after elective major abdominal surgery in elderly patients: A cohort study	10.1016/j.ijsu.2019.09.011	43	A single-center cohort study	POD is a frequent complication after major abdominal surgery in the elderly, especially in octogenarians and after-open procedures
Indrakusuma et al. (2015) (24)	Netherlands	Evaluation of preoperative geriatric assessment of elderly patients with colorectal carcinoma. A retrospective study	10.1016/j.ejso.2014.09.005	37	Retrospective cohort and match-control study	While the DOG patients were significantly more at risk for postoperative complications, the DOG patients had comparable postoperative outcomes as their matched controls
Breugom et al. (2016) (25)	Netherlands	Between the Most Frequent Complications After Surgery for Stage I-III Colon Cancer and Short-Term Survival, Long-Term Survival, and Recurrences	10.1245/s10434-016-5226-z	32	Clinical study	The results demonstrate the serious impact of the most frequent complications after surgery for colon cancer on short-term and long-term outcomes
Mokutani et al. (2016) (26)	Japan	Prediction of Postoperative Complications Following Elective Surgery in Elderly Patients with Colorectal Cancer Using the Comprehensive Geriatric Assessment	10.1159/000446709	31	Clinical study	The CGA was a useful predictor of postoperative complications in elderly patients when administered before surgery for CRC

POD, postoperative delirium; LSMM, low skeletal muscle mass; DOG, Dutch acronym; CGA, comprehensive geriatric assessment; CRC, complications of colorectal cancer.

**Figure 4:**
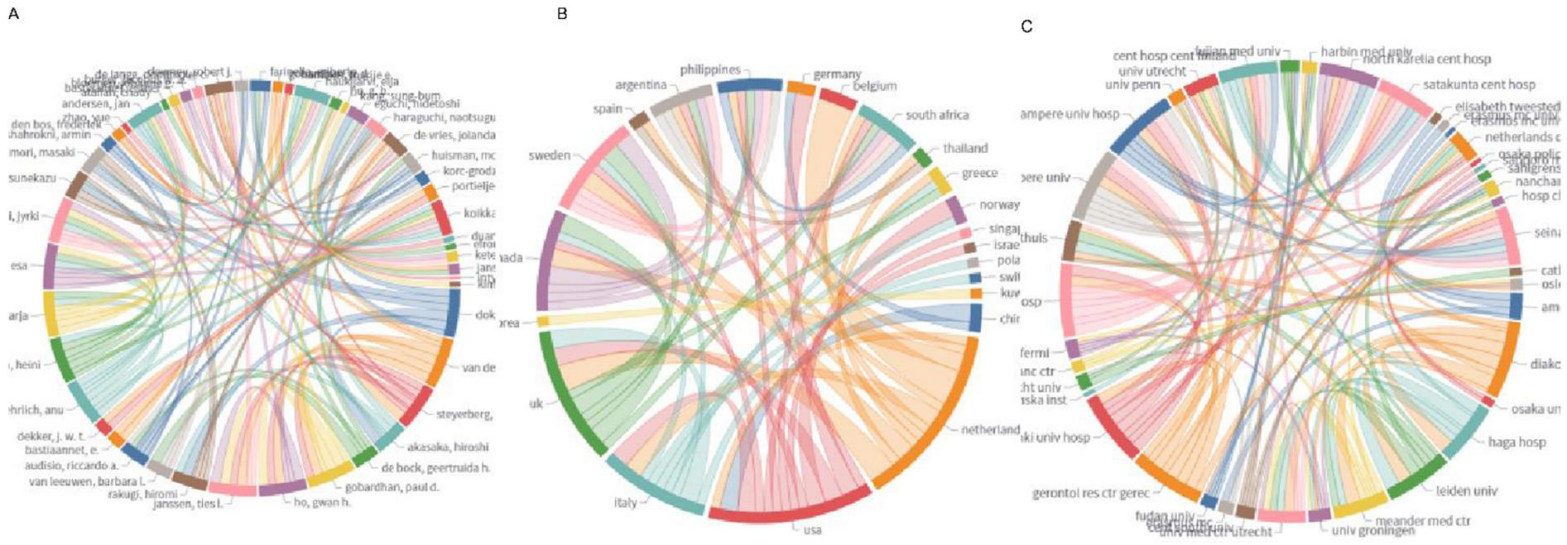
Analysis of collaboration matrix in PNDs research for colorectal cancer surgery. **(A)** Collaboration authors; **(B)** collaboration country; **(C)** collaboration organization.

Furthermore, a collaboration analysis on the authors, country, and organization was performed as shown in Figures 4A–C. Figure 4A revealed that Isolated network centered on Doki Yuichiro and Rakugi Hiromi (Osaka University) with high internal collaboration (4–6 links) but zero international ties. High-density hub around van der Laan Lijckle (Amphia Hospital), linking surgeons (Gobardhan Paul), methodologists (Steyerberg Ewout), and geriatric researchers (Janssen Ties). Moreover, Figure 4B demonstrated strong intra-European links with Belgium (4), UK (3), Sweden (2), and Italy (2), and minimal outreach to Asia (only 2 ties with China). Figure 4C illustrated the Amphia, Diakonessen, Haga, and Leiden form a tight cluster (4–5 links), enabling integrated geriatric-surgical research. Limited academic ties (e.g., low links to University Medical Center Utrecht). Those collaboration networks reveal three critical patterns: first, Dutch leadership in clinically integrated research (hospital-university synergy) drives PND prehabilitation studies but lacks neurobiological expertise; second, Asia's high-volume, low-collaboration trap: China/Japan/Korea produce 60 publications (34% of global output) yet operate in isolation, explaining low citation impact and stagnant translation; third, No significant collaborations involve anesthesiologists or neurologists across all matrices, perpetuating a narrow surgical focus on PNDs while ignoring mechanisms (e.g., neuroinflammation biomarkers). This fragmentation hinders the field from evolving beyond delirium management to neurocognitive prevention*.*

### 3.2 Systematic analysis

Recent clinical studies (2014–2025) demonstrate significant progress in mitigating PND among elderly patients undergoing colorectal cancer surgery, with pharmacological and multimodal interventions showing promising efficacy (3, 4, 7, 27–34) As summarized in Table 4, 11 trials predominantly from China and Egypt (total 1,308 patients, mean age 56–76 years) reveal several research trends. First, studies investigated the pharmacological prophylaxis reduces PND incidence, including melatonin prophylaxis decreased POD by 20% (56% vs*.* 36%) (27), dexmedetomidine-enhanced regional anesthesia (4, 7) lowered PND/POCD rates by 7%–25% through anti-inflammatory effects (reduced IL-6/TNF-α), and also facilitate recovery (31), and intranasal insulin can reduce POCD by 25.4% (38.7% vs. 13.3%) via neuroinflammation modulation (34). Second, the anesthetic and surgical innovation yield dual benefits, such as, remimazolam outperformed propofol in PND prevention (cognitive assessments) (3), Jia et al., revealed that fast-track surgery reduced POD by 9.5% (12.9% vs. 3.4%) while accelerating gastrointestinal recovery (28), Sun et al., revealed preemptive multimodal analgesia can reduce the incidence of POGD and POD in elderly patients (32), Wu et al., also found that goal-directed fluid therapy appears to reduce the incidence of early POCD, accelerate postoperative recovery (33), active transcranial direct current stimulation (tDCS) decreased POD by 17.3% (25.5% vs. 8.2%) and improved pain/anxiety metrics (29). Finally, critical risk factor includes advanced age (>60 years), ASA status II–III, and surgical duration have significant impact on PND. Notably, inspired oxygen fraction (FiO₂) did not significantly affect POD (Lin et al.), challenging conventional oxygenation practices (30). Moreover, Supplementary Table 1 summarizes the risk of bias among the included RCTs, revealing that over 80% of the studies were assessed as having a low risk of bias (3, 4, 7, 27, 29–31, 33, 34), with one study exhibiting an unclear risk of bias (28), and another demonstrating high risk of bias (32).

**Table 4:** The details of included clinical studies on the perioperative neurocognitive disorder in patients undergoing colorectal cancer surgery.

Study	Country	Mean age	Male/female	ASA	Surgery	POD/POCD/PND incidence	Conclusions
Elbakry et al. (2024) (27)	Egypt	76 ± 4.8 vs. 76.6 ± 6	35/15 vs. 42/8	I–III	Elective colorectal cancer surgeries	POD: 28 (56%) vs. 18 (36%)	The prophylactic administration of melatonin may decrease the incidence of POD in elderly patients undergoing colorectal surgeries under general anesthesia
Jia et al. (2014) (28)	China	74.78 ± 4.01 vs. 75.66 ± 4.18	70/46 vs. 76/41	NA	Open colorectal surgery	POD: 15 (12.9%) vs. 4 (3.4%)	Compared to traditional perioperative management, fast-track surgery decreases the LOS, facilitates the recovery of bowel movement, and reduces occurrence of POD and other complications in elderly patients with colorectal carcinoma
Li et al. 2024 (29)	China	63.5 ± 10.3 vs. 63.5 ± 11.8	60/38 vs. 64/34	II–III	Laparoscopic colorectal cancer surgery	POD:8.2% vs. 25.5%	Active tDCS was also associated with better anxiety scores, pain levels, and sleep quality as well as reduced POD and frailty
Lin et al. 2021 (30)	China	71.39 ± 2.80 vs. 71.45 ± 3.85	NA	I–III	Laparoscopic radical gastrectomy (radical resection of colon cancer, rectal cancer only)	POD: 64 (23.8%) vs. 58 (18.35%)	The incidence of POD was not significantly affected by different FiO2 and the incidence of postoperative atelectasis was decreased at low FiO2
Liu et al. 2020 (31)	China	69.6 ± 4.4 vs. 69.3 ± 4.4 vs. 68.5 ± 4.2 vs. 68.6 ± 3.9	15/9 vs*.* 14/10 vs*.* 15/9 vs. 13/11	I–II	Radical resection for colorectal cancer	POCD: 20.8% vs. 29.2% vs. 12.5% vs. 29.2%	A combination of intraoperative dexmedetomidine infusion and epidural blockade could mitigate pain after surgery, improve cognitive dysfunction in early surgery, and facilitate recovery
Liu et al. 2025 (3)	China	73.9 ± 6.3 vs*.* 69.7 ± 4.4 vs*.* 72.3 ± 5.6 vs. 71.5 ± 5.2	14/16 vs*.* 12/16 vs*.* 13/17 vs*.* 15/14	II–III	Laparoscopic colorectal surgery	PND: evaluated using MMSE (Mini-Mental State Examination) and MoCA (Montreal Cognitive Assessment)	Compared to propofol, remimazolam at medium and high doses can reduce the incidence of PND in elderly patients
Sun et al. 2024 (32)	China	73.14 ± 6.87 vs*.* 73.86 ± 7.12	40/27 vs. 38/28	I–III	Laparoscopic colorectal surgery	POD: 6 (8.96%) vs. 16 (24.24%)	Preemptive multimodal analgesia can reduce the incidence of POGD and POD in elderly patients undergoing laparoscopic gastrocolic surgery, improve the recovery process of postoperative gastrointestinal function
Wu et al. (2024) (33)	China	66.37 ± 4.85 vs. 64.87 ± 5.12	23/17 vs. 21/19	II–III	Elective laparoscopic radical resection of colorectal cancer	POCD: 6 (15%) vs. 12 (30%)	GDFT appears to reduce the incidence of early POCD, accelerate postoperative recovery, and enhance overall prognosis.
Yang et al. 2024 (4)	China	63.61 ± 7.08 vs*.* 62.16 ± 6.68	50/34 vs*.* 58/27	NA	Radical laparoscopic colorectal cancer surgery	PND: 6h:17.9% vs*.* 7.1%, 24h:14.3%vs*.* 4.7%, 30-day:11.9% vs. 3.5%	Dexmedetomidine added to ropivacaine for TAPB can reduce the incidence of PND in the first 24 h after surgery and on the 30th postoperative day, which may be related to reduce the consumption of general anesthetics and provide satisfactory postoperative analgesia
Zhang et al. (2024) (34)	China	70.7 ± 4.2 vs. 69.9 ± 3.9	20/12 vs*.* 22/10	I–III	Laparoscopic radical resection of colorectal cancer	POCD: 4 (13.3%) vs. 12 (38.7%)	Intranasal insulin may decrease the risk of POCD and inhibit the elevated serum IL-6, TNF-α, and S100β levels
Zhang et al. (2025) (7)	China	56.30 ± 11.40 vs*.* 55.90 ± 12.10	34/26 vs. 32/28	I–III	Radical resection for colorectal cancer	POCD: Evaluated using MoCA	Dexmedetomidine plus ropivacaine-enabled TAPB reduces POCD and inflammatory/stress hormone levels, and significantly improves the postoperative analgesic effect

The data are presented as means ± SD, median (interquartile range), or *n* (%) depending on type and distribution. POD, postoperative delirium; POCD, postoperative cognitive dysfunction; PNDs, perioperative neurocognitive disorders; LOS, length of hospital stay; ASA, American society of anesthesiologists physical status classification; tDCS, transcranial direct current stimulation; FiO2, fraction of inspired oxygen; MMSE, mini-mental state examination; MoCA, montreal cognitive assessment; POGD, postoperative gastrointestinal dysfunction; GDFT, goal-directed fluid therapy; TAPB, transversus abdominis plane block.

Moreover, Supplementary Table 2 summarizes the variability in PND diagnostic criteria and assessment timelines across the included RCTs. Of the 11 RCTs, two studies employed the Mini-Mental State Examination (MMSE) with cutoff scores ranging from 24 to 27/30 (4, 31); two utilized the Montreal Cognitive Assessment (MoCA) with a standardized cutoff score of 26/30 (7, 33); one combined both the MMSE and MoCA for comprehensive cognitive evaluation (3); and three specifically adopted the Confusion Assessment Method (CAM) for the diagnosis of POD (29, 30, 32). One additional study integrated three assessment tools: the Nursing Delirium-Screening Score (NU-DESC) (27), the Delirium Rating Scale-Revised-98 (DRS-R-98) (28), and criteria from the International Study Group of Postoperative Cognitive Dysfunction (ISPOCD) (34). Notably, only one study assessed the severity of POD, utilizing the Memorial Delirium Assessment Scale (MDAS) (30).

Regarding assessment timelines, evaluations spanned the early postoperative period (6 h), short-term follow-up (Days 1–7) (3, 7, 27–34), and intermediate follow-up (30 days) (4). Critically, no studies evaluated long-term cognitive outcomes (i.e., >30 days)—a gap that constrains the ability to draw robust conclusions regarding chronic PND in patients undergoing colorectal cancer surgery.

## 4 Discussion

This review employs an integrative bibliometric-systematic methodology to interrogate the mechanistic and clinical dimensions of PND in colorectal cancer surgery populations. Bibliometric analysis reveals significant geographic clustering in PND research. The Netherlands dominated institutional productivity (7/10 top institutions) and high-impact references (6/10 top-cited studies). Dutch cohorts pioneered risk stratification models, identifying age (18), low skeletal muscle mass (22), and open surgery (10) as key predictors, whereas Chinese teams focused on interventional innovations (e.g., dexmedetomidine, tDCS) (4, 7, 29, 31). Although these references reflect the research trends and hotspots, the limitations of these literatures are also obvious: they are basically single-center retrospective studies with limited sample sizes. Furthermore, the conclusions focus on the description of risk factors, and there is a lack of evidence-based support for prevention strategies. Cross-cultural validation is insufficient, such as whether the DOG assessment tool developed in the Netherlands is suitable for Asian populations. Future research should turn to multicenter RCTs to validate prehabilitation protocols, explore neuroinflammatory biomarkers, and compare the effects of different anesthesia techniques. In terms of clinical practice, there is a need to integrate CGA assessment and nutritional interventions, especially for screening patients with sarcopenia.

The current systematic review revealed that the lack of standardized PND definitions impedes cross-study synthesis. First, among the 11 included studies, evaluation timelines varied from 6 h postoperatively (4) to 30 days, neglecting long-term cognitive trajectories, thereby limiting insights into chronic PND. Second, 90% of trials targeted patients >63 years (3, 4, 27–34), excluding younger high-risk populations and restricting the generalizability of findings to broader surgical cohorts (7).

Additionally, non-pharmacological interventions were underrepresented, with only two studies investigating such strategies (28, 29). Specifically, Li et al. evaluated neuromodulation via tDCS, reporting a 17.3% reduction in POD-an efficacy comparable to that of pharmacotherapies (29). Jia et al., meanwhile, compared fast-track surgery with traditional perioperative management, demonstrating that fast-track protocols shortened hospital stays, accelerated bowel function recovery, and reduced the incidence of POD and other complications in elderly patients with colorectal carcinoma (28). Notably, other non-pharmacological approaches (e.g., cognitive training, multidisciplinary rehabilitation, nutritional optimization) have been shown to mitigate PND in related surgical contexts (35–37), but remain unstudied in this specific population. Another critical gap is the absence of cost-effectiveness analyses for interventions such as CGA, despite evidence from Mokutani et al. that CGA reduces postoperative complications by 30% in elderly patients (26).

Mechanistically, studies have shown that dexmedetomidine reduces levels of IL-6 and TNF-α by 40%–60% (4, 7), which correlated with a 14.4%–25% reduction in PND risk. Similarly, intranasal insulin lowered serum IL-6, TNF-α, and S100β levels by 35% and reduced POCD by 25.4% (34). While elevations in these inflammatory markers (IL-6, TNF-α, S100β) are associated with PND—supporting a plausible (though not definitive) mechanistic role (35). It is important to note that cytokine reduction alone is insufficient for PND prevention (37). Further, two factors were identified as significant modifiers of PND risk: surgical approach and goal-directed fluid therapy (28, 33).

Despite robust evidence supporting intervention efficacy, four key barriers hinder clinical translation. First, the optimal dose of dexmedetomidine for PND prevention remains undefined. Second, implementation of tDCS and CGA requires specialized personnel and training, which may limit accessibility. Third, while S100β and IL-6 show promise as potential biomarkers, no PND-specific biomarkers have been clinically validated. Finally, although fast-track surgery reduces POD by 9.5%, its synergistic effects with pharmacological agents (e.g., melatonin) remain unexplored.

### 4.1 Limitations of the current study

While this bibliometric-systematic review provides a comprehensive synthesis of PND in colorectal cancer surgery, three methodological constraints merit acknowledgment. First, significant geographic bias limits the generalizability of findings. Over 80% of interventional trials originated from China, while high-impact risk-factor studies were predominantly from the Netherlands. This skew may overlook region-specific practices affecting PND outcomes. Second, methodological heterogeneity in PND assessment undermines cross-study comparisons. Third, diagnostic criteria and assessment timeline heterogeneity may hinder cross-study synthesis. Last but not least, the systematic exclusion of key populations and interventions restricts clinical applicability.

### 4.2 Future direction

Building on the limitations and gaps identified in this review, future research on PND in colorectal cancer surgery should prioritize the following targeted areas to advance clinical practice and scientific understanding.

## 5 Conclusions

PND in colorectal cancer surgery is mitigated by multimodal strategies. While dexmedetomidine and fast-track, surgery is supported by the best available RCT evidence, they should be considered conditional recommendations rather than definitive standards. These reduce PND incidence by 15%–25% through neuroinflammation suppression and optimized analgesia. Persistent challenges include geographic bias (80% trials from China), heterogeneous assessment methods, and neglect of younger populations. Future research must standardize PND definitions using MoCA/MMSE thresholds, validate tDCS in multicenter trials, and explore long-term cognitive outcomes. Integrating comprehensive geriatric assessments globally remains essential for equitable PND prevention.

## Data Availability

The original contributions presented in the study are included in the article/Supplementary Material, further inquiries can be directed to the corresponding author.
