# Supplementary material for: Perioperative neurocognitive disorder in colorectal cancer surgery: a systematic review of incidence, mechanisms, and interventions
Source: Front Surg. 2025 Nov 18;12:1698597. doi: 10.3389/fsurg.2025.1698597 (PMC12669154; doi:10.3389/fsurg.2025.1698597)
Supplement: Supplementary file 1 [file Table1.doc]

Supplementary Table-1: Search strategy for different data-base.

2025-07-22,web of science,147

| #1(((((TS=(Postoperative delirium)) OR TS=(Postoperative Cognitive Complications)) OR TS=(Postoperative neurocognitive disorders)) OR TS=(perioperative neurocognitive disorders)) OR TS=(postoperative cognitive dysfunction)) OR TS=(postoperative cognitive decline) |
| --- |
| #2TS=(colorectal cancer surgery) |
| #3 #1 and #2 |

2025-07-22,pubmed,68

| #1 **((((((((("Postoperative Cognitive Complications"[Mesh]) OR (Postoperative Cognitive Complication[Title/Abstract])) OR (Postoperative Cognitive Decline[Title/Abstract])) OR (Postoperative Cognitive Dysfunction[Title/Abstract])) OR (Postoperative Cognitive Disorders[Title/Abstract])) OR (Postoperative Cognitive Disorder[Title/Abstract])) OR (Postoperative delirium[Title/Abstract])) OR (Postoperative neurocognitive disorders[Title/Abstract])) OR (perioperative neurocognitive disorders[Title/Abstract])) OR (postoperative cognitive dysfunction[Title/Abstract])** |
| --- |
| #2**((colorectal cancer surgery[Title/Abstract]) OR (colorectal surgery[Title/Abstract])) OR (colorectal cancer[Title/Abstract])** |
| #3 #1 and #2 |

2025-07-22,Embase,128

| #1'postoperative delirium' OR 'postoperative cognitive dysfunction' OR 'postoperative cognitive complications' OR 'postoperative neurocognitive disorders' OR 'perioperative neurocognitive disorders' OR 'postoperative cognitive decline' |
| --- |
| #2 'colorectal cancer surgery' OR 'colorectal surgery' |
| #3#1 and #2 |
